# Excess US Deaths Before, During, and After the COVID-19 Pandemic

**DOI:** 10.1001/jamahealthforum.2025.1118

**Published:** 2025-05-23

**Authors:** Jacob Bor, Rafeya V. Raquib, Elizabeth Wrigley-Field, Steffie Woolhandler, David U. Himmelstein, Andrew C. Stokes

**Affiliations:** 1 Department of Global Health, Boston University School of Public Health, Boston, Massachusetts; 2 Department of Epidemiology, Boston University School of Public Health, Boston, Massachusetts; 3 Minnesota Population Center, University of Minnesota, Minneapolis; 4 Department of Sociology, University of Minnesota, Minneapolis; 5 Hunter College, City University of New York, New York, New York; 6 Cambridge Health Alliance/Harvard Medical School, Cambridge, Massachusetts

## Abstract

This cohort study examines trends in excess deaths in the US before, during, and after the COVID-19 pandemic.

## Introduction

Mortality rates decreased more slowly in the US than in other high-income countries (HICs) between 1980 and 2019,^1^ resulting in growing numbers of excess US deaths compared with other HICs.^1,2,3,4^ We assessed trends in excess US deaths before (1980-2019), during (2020-2022), and after (2023) the acute phase of the COVID-19 pandemic.

## Methods

This cross-sectional study was deemed exempt from review and informed consent by the Boston University Institutional Review Board because no human participants were included. We followed the STROBE reporting guideline.

We obtained all-cause mortality data for the US and 21 other HICs from the Human Mortality Database from January 1980 to December 2023.^5^ For each year, we computed age-specific mortality rates for the US and the population-weighted average of other HICs. We then calculated the number of US deaths that would have been expected each year had the US population experienced the age-specific mortality rates of other HICs. We computed ratios of observed-to-expected US deaths. We then computed numbers of excess deaths attributable to the US mortality disadvantage by taking the difference between observed and expected US deaths. We stratified by age. Finally, we fit a linear regression model to assess whether the number of excess US deaths in 2023 differed from the 2014-2019 prepandemic trend (eMethods in Supplement 1). Analyses were conducted with Stata/MP, version 18.0 (StataCorp LLC), and R, version 4.42 (R Project for Statistical Computing).

## Results

Our analysis encompassed 107 586 398 deaths in the US and 230 208 265 deaths in other HICs from 1980 to 2023. We estimate that 14 735 913 excess deaths occurred in the US in this period compared with other HICs. US mortality rose rapidly in 2020 and 2021 during the pandemic, then declined in 2022 and 2023. The pandemic-era mortality surge was less pronounced in other HICs (Figure, A).

**Figure.**
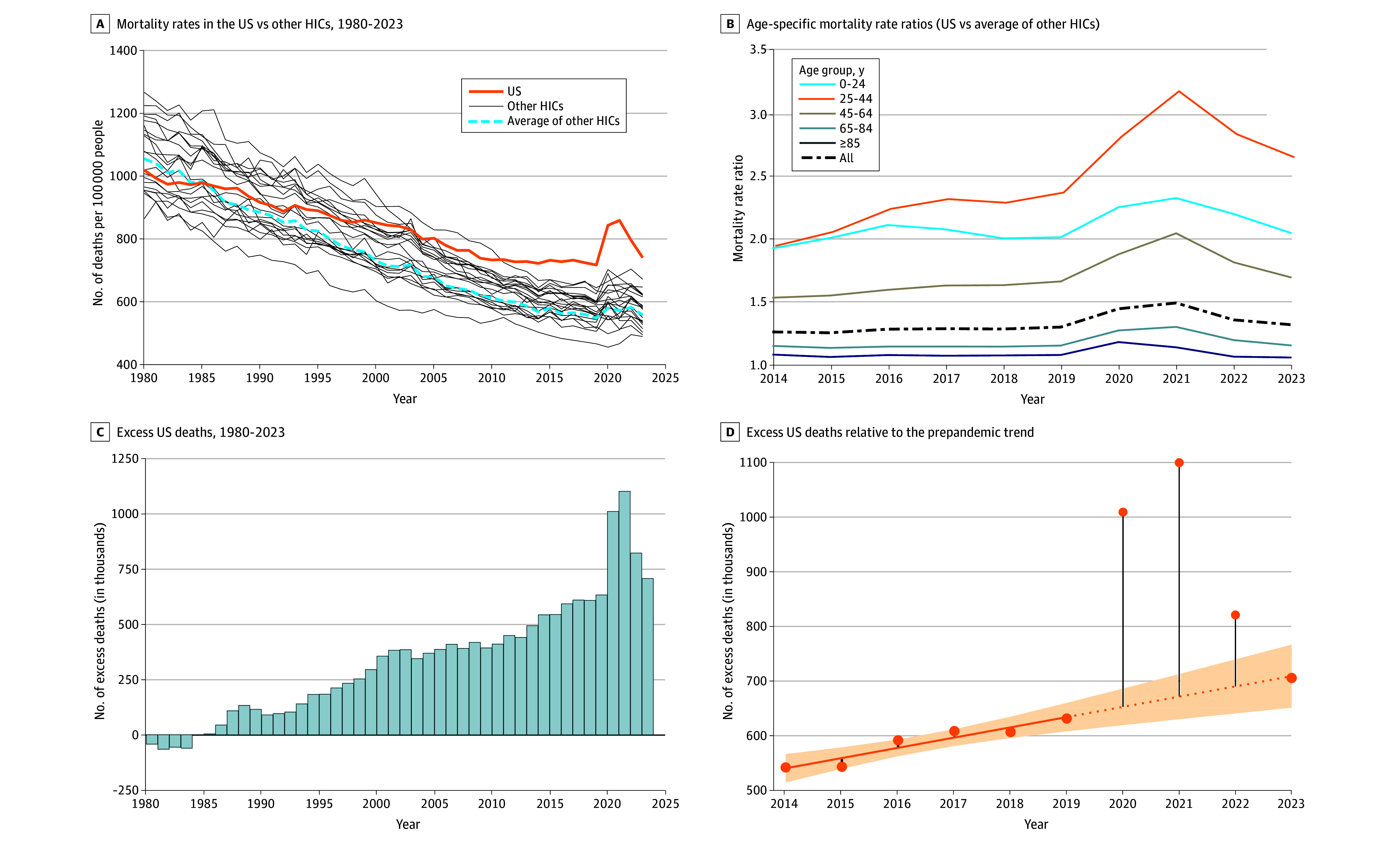
Mortality Rates, Mortality Rate Ratios, and Excess US Deaths Attributable to the US Mortality Disadvantage Relative to Other High-Income Countries (HICs) A, Trends in US mortality rates, mortality rates of other HICs, and average mortality rates in other HICs standardized to the US age distribution in each year (1980-2023). B, Age-specific mortality rate ratios comparing US mortality rates to the average of other HICs (2014-2023). C, Excess deaths attributable to the US mortality disadvantage (1980-2023). D, Linear extrapolation of the prepandemic trend in excess deaths over the period from 2020 to 2023. B and D start in 2014 to enable visualization of trends immediately before, during, and after the acute phase of the COVID-19 pandemic. The comparison set of HICs included Australia, Austria, Belgium, Canada, Denmark, Finland, France, Germany, Iceland, Ireland, Italy, Japan, Luxembourg, the Netherlands, New Zealand, Norway, Portugal, Spain, Sweden, Switzerland, and the United Kingdom. In panel D, the solid orange line shows the linear regression fit for 2014-2019; the dotted orange line extrapolates this trend through 2020-2023; the shaded area indicates 95% CIs; and the vertical black lines indicate deviations of excess US deaths from what would be expected based on the prepandemic trend.

Relative differences between the US and other HICs widened before and during the pandemic, particularly among younger adults, before contracting in 2022 and 2023. Age-standardized mortality rate ratios comparing the US with the average of other HICs were 1.20 in 2010, 1.28 in 2019, 1.46 in 2021, and 1.30 in 2023 (Table). In 2023, mortality among US adults aged 25-44 years was 2.6 times higher than in other HICs (Figure, B).

**Table.** Observed Deaths, Expected Deaths, Mortality Rate Ratios, and Excess Deaths Attributable to the US Mortality Disadvantage, 1980-2023

Year	All ages				Children and adults (aged 0-64 y)				Older adults (aged ≥65 y)			
	No. of US deaths		Mortality rate ratio	No. of excess US deaths (% of observed)	No. of US deaths		Mortality rate ratio	No. of excess US deaths (% of observed)	No. of US deaths		Mortality rate ratio	No. of excess US deaths (% of observed)
	Observed	Expected			Observed	Expected			Observed	Expected		
1980	1 989 837	2 031 945	0.98	−42 109 (−2.1)	647 614	522 254	1.24	125 360 (19.4)	1 342 222	1 509 691	0.89	−167 469 (−12.5)
1990	2 148 467	2 058 809	1.04	89 658 (4.2)	606 088	461 031	1.31	145 057 (23.9)	1 542 379	1 597 778	0.97	−55 399 (−3.6)
2000	2 403 399	2 048 411	1.17	354 987 (14.8)	603 345	447 098	1.35	156 247 (25.9)	1 800 054	1 601 313	1.12	198 741 (11.0)
2010	2 468 426	2 059 256	1.20	409 170 (16.6)	670 064	456 743	1.47	213 322 (31.8)	1 798 362	1 602 513	1.12	195 849 (10.9)
2019	2 854 826	2 223 579	1.28	631 247 (22.1)	737 398	419 815	1.76	317 583 (43.1)	2 117 428	1 803 764	1.17	313 664 (14.8)
2020	3 383 749	2 375 380	1.42	1 008 369 (29.8)	874 271	436 569	2.00	437 702 (50.1)	2 509 479	1 938 812	1.29	570 667 (22.7)
2021	3 464 260	2 365 452	1.46	1 098 808 (31.7)	969 489	441 582	2.20	527 908 (54.5)	2 494 771	1 923 871	1.30	570 900 (22.9)
2022	3 279 915	2 459 519	1.33	820 396 (25.0)	853 052	432 049	1.97	421 003 (49.4)	2 426 863	2 027 470	1.20	399 393 (16.5)
2023	3 081 628	2 376 297	1.30	705 331 (22.9)	777 813	419 866	1.85	357 947 (46.0)	2 303 815	1 956 431	1.18	347 384 (15.1)

Excess deaths attributable to the US mortality disadvantage peaked at 1 008 369 in 2020 and 1 098 808 in 2021, then declined to 820 396 in 2022 and 705 331 in 2023. These numbers followed 4 decades of rising excess deaths, reaching 631 247 in 2019 (Figure, C and Table). In 2023, excess US deaths accounted for 22.9% of all deaths and 46.0% of deaths among US residents younger than 65 years (Table).

Regression analysis demonstrated that the rising trend in excess US deaths before 2020 continued during the pandemic. Excess deaths in 2023, although lower than in 2020 to 2022, were higher than in 2019 and consistent with the slope established from 2014 to 2019 (Figure, D).

## Discussion

Between 1980 and 2023, the total number of excess US deaths reached an estimated 14.7 million.^1^ Although excess deaths per year peaked in 2021, there were still more than 1.5 million during 2022 to 2023. In 2023, excess death rates remained substantially higher than prepandemic rates. The rising trend from 1980 to 2019 appears to have continued during and after the pandemic, likely reflecting prepandemic causes of death, including drug overdose, firearm injury, and cardiometabolic disease.^6^ These deaths highlight the continued consequences of US health system inadequacies, economic inequality, and social and political determinants of health.^1,2,3,5^

Study limitations include potential sensitivity to choice of comparison countries, use of provisional data for some countries in 2023, and lack of stratification to investigate differences by sex, race and ethnicity, and socioeconomic status. Our results suggest that policy solutions may be found in the experiences of other HICs. Future research is needed to identify the specific causes of the widening US mortality disadvantage and opportunities for intervention.

## Notes

Supplement 1.
eMethods

eReferences

Supplement 2.
Data Sharing Statement
