# Error in Funding/Support

**DOI:** 10.1001/jamahealthforum.2025.2950

**Published:** 2025-07-11

In the Research Letter titled “Excess US Deaths Before, During, and After the COVID-19 Pandemic,”^1^ published May 23, 2025, the number of the grant awarded to Dr Wrigley-Field was inadvertently omitted from the Funding/Support section of the Article Information. This article has been corrected.
