# Supplementary material for: Excess US Deaths Before, During, and After the COVID-19 Pandemic
Source: JAMA Health Forum. 2025 May 23;6(5):e251118. doi: 10.1001/jamahealthforum.2025.1118 (PMC12102698; doi:10.1001/jamahealthforum.2025.1118)
Supplement: Supplement 1. — eMethods eReferences [file jamahealthforum-e251118-s001.pdf]

Supplemental Online Content

Bor J, Raquib RV, Wrigley-Field E, Woolhandler S, Himmelstein DU, Stokes AC. Excess US deaths before, during, and after the COVID-19 pandemic. *JAMA Health Forum*. 2025;6(5):e251118. doi:10.1001/jamahealthforum.2025.1118

eMethods

eReferences

This supplemental material has been provided by the authors to give readers additional information about their work.

© 2025 Bor J et al. *JAMA Health Forum*.

eMethods

Data

Our analysis used data from the Human Mortality Database (HMD) from 1980 to 2023. The HMD project compiles national vital registry and census data into multiple data products. The first data product we used – the standard HMD “long series” – presents age-specific mortality data (deaths and death rates) for each country-year. While the “long series” contained data through 2023 for some countries, other countries were missing data for 2023. In those case we used data from a second HMD data product – the Short-Term Mortality Fluctuations (STMF) database. The STMF was launched during the COVID-19 pandemic to provide more timely mortality data to researchers and includes weekly death counts from the mid-2010s to present (data are updated regularly). A limitation of the STMF is that age categories are not standardized across countries, with the exception of a few broad categories that HMD provides (e.g., 15-65 years). For our analysis, we used the original input data from the STMF database. Data were considered provisional for some countries in 2023. We accessed both databases on February 18, 2025. The HMD “long series” was last updated on February 4, 2025, and the STMF was last updated February 17, 2025.

Comparison countries

The 21 comparison countries – other wealthy nations (OWNs) – included Australia, Austria, Belgium, Canada, Denmark, Finland, France, Germany, Iceland, Ireland, Italy, Japan, Luxembourg, The Netherlands, New Zealand, Norway, Portugal, Spain, Sweden, Switzerland, and the United Kingdom. We used identical inclusion criteria to our prior study for purposes of direct comparability.[1] We included countries with available data in the HMD for the period 1980 to 2022 or later and a 2021 gross domestic product exceeding US $24,000 per capita. We excluded former Soviet / Eastern Bloc countries due to major mortality differences occurring with the fall of the Soviet Union. All countries were observed through 2023, with the exception of Ireland, which lacked data for 2023 in both the HMD “long series” and STMF. We imputed values for Ireland in 2023 using the values and slope from 2021 and 2022. Sensitivity analyses showed our findings were robust to other imputation approaches, including carrying forward the 2022 value and imputing a 2023 value based on the proportional change in the United Kingdom.

Periodization

Excess deaths are shown beginning in 1980 to show their growth over the full period of US disadvantage. In prior work, we showed excess deaths starting in 1933, when data first became available, and show that excess deaths turn positive starting around 1980.[1] Our regression analysis was based on 2014-2019, in line with prior work on U.S. excess mortality.[2] Sensitivity analyses revealed that inferences were robust to the choice of starting year (2010-2017).

Data extraction and manipulation

This analysis used annual age-specific mortality rates (ASMRs), death counts, and exposure time (e.g., person-years) from the HMD “long series” for 5-year age groups. When these values were missing from the “long series” in years from 2020 to 2023, we extracted the original input data from the STMF database and aggregated to annual death counts for 5-year age groups. In

© 2025 Bor J et al. *JAMA Health Forum*.

cases where a country did not present 5-year age groups for a particular age range, we used the distribution of deaths from the last year available in the HMD long series (e.g., deaths at ages 20-29 in 2023 were allocated to ages 20-24 and 25-29 using the distribution of deaths in the last year where 5-year age groups were available). We obtained denominators for the death counts by fitting a linear trend to the 2016-2020 exposure data from the HMD "long series" for each 5-year age group in each country (e.g., Norway, population aged 20-24 years in 2021 was imputed based on a linear fit for Norway's population aged 20-24 years in 2016-2020).

Our final dataset included ASMRs for the U.S. and the 21 OWNs over 5-year age groups for each year from 1980 through 2023. We aggregated the age categories to 5 larger age groups (0-24, 25-44, 45-64, 65-84, 85+) and 2 larger age groups (0-64, 65+) for age-stratified analyses. We then calculated the average ASMRs of OWNs for each 5-year age group and year, weighting countries by their populations, following our prior work.[1] The population weighted average of OWNs reflects the average experience of a person residing in an OWN.

Calculation of excess deaths comparing the U.S. to OWN

We calculated the expected number of U.S. deaths in each year if the U.S. had ASMRs equal to the average of OWNs. To do so, we multiplied the U.S. population distribution in 5-year age groups (from the HMD "long series" annual exposure data) by the population-weighted average ASMRs for OWNs. Excess deaths were calculated by taking the difference between observed U.S. deaths and expected U.S. deaths based on OWN ASMRs. We also compared observed ASMRs each year in the U.S. to the observed ASMRs in OWNs by calculating ASMR ratios comparing the U.S. to OWN for both the 5 larger and 2 larger age categories.

eReferences

1. Bor J, Stokes AC, Raifman J, et al. Missing Americans: Early death in the United States-1933-2021. *PNAS Nexus*. 2023;2(6):gad173.

2. Woolf SH, Chapman DA, Sabo RT, Zimmerman EB. Excess Deaths From COVID-19 and Other Causes in the US, March 1, 2020, to January 2, 2021. *JAMA*. 2021;325(17):1786-1789.

© 2025 Bor J et al. *JAMA Health Forum*.
