# Supplementary material for: Excess US Deaths Before, During, and After the COVID-19 Pandemic
Source: JAMA Health Forum. 2025 May 23;6(5):e251118. doi: 10.1001/jamahealthforum.2025.1118 (PMC12102698; doi:10.1001/jamahealthforum.2025.1118)
Supplement: Supplement 2. — Data Sharing Statement [file jamahealthforum-e251118-s002.pdf]

Data Sharing Statement

Bor. Excess US Deaths Before, During, and After the COVID-19 Pandemic. *JAMA Health Forum*. Published May 23, 2025. doi:10.1001/jamahealthforum.2025.1118

Data
Data available: Yes
Data types: Data (not involving human participants)
How to access data: Data are posted at https://osf.io/u6s2x/.
When available: With publication

Supporting Documents
Document types: Statistical/analytic code
How to access documents: Code is posted at https://osf.io/u6s2x/.
When available: With publication

Additional Information
Who can access the data: Data and code are publicly available.
Types of analyses: Data and code are publicly available for any and all uses.
Mechanisms of data availability: Data are publicly available on an OSF repository (https://osf.io/u6s2x/) and are freely downloadable without any application or approval.
